# Supplementary material for: Bridging the gap in pneumonia prevention: Qualitative insights on vaccine implementation from health leaders in middle-income countries
Source: PLOS Glob Public Health. 2025 Apr 23;5(4):e0004473. doi: 10.1371/journal.pgph.0004473 (PMC12017571; doi:10.1371/journal.pgph.0004473)
Supplement: S1 Text — (DOCX) [file pgph.0004473.s001.docx]

**S1 Text: Interview Guide: Policymaker perspectives on childhood pneumonia prevention and pneumococcal conjugate vaccine (PCV) introduction**

*Purpose*: The purpose of this tool is to explore perceptions of policymakers on pneumococcal disease burden, specifically childhood pneumonia prevention, perceptions of pneumococcal severity and perceptions of pneumococcal susceptibility, treatment and prevention choices. We will also gather their thoughts on factors likely to influence PCV introduction, rollout, uptake and acceptance on a national scale. The target policy makers will include immunization program managers, Ministry of Health (MoH) officials, and members of implementation organizations such as the United Nations Children's Fund (UNICEF) and Save the Children.

*Directions*:

- Please ensure that these questions are read out loud to the participant, and their responses are documented.
- Please document as many responses as possible for the open-ended questions. If additional notes are needed, please refer back to the recording captured.
- These questions (in BOLD) are designed to maintain the conversation.
- After each question, in brackets, is a list of probes that may or may not be used to prompt caregivers to continue their conversation.
- You do not need to use every probe in the guide; the probes are there to remind you to consider those dimensions when listening to the policymaker.
- We need to hear from the policymaker as much as possible. It is important not to suggest answers or ask questions in a way that leads the policymaker to a particular answer.

**Please read text below to policymaker**

The purpose of this interview is to learn about your perceptions of pneumonia and pneumococcal disease, your experiences with vaccine introduction, and your perceptions of strategies to strengthen vaccine delivery and uptake. As we discussed during the consent process, I will be taking notes on what you say, and I would like to record the interview so that I can make sure that I don’t miss anything you say. Your name will not be recorded. The recording will not be shared with anyone outside of the study team and will be destroyed after the study is complete.

Demographic Overview Questions

1. **Please state your current designation and the name of your organization:**

| - 1. Designation: _____________________________ | - 1. Organization:  1. Minstry of Health 2. UNICEF 3. Save the Children 4. Other, specify: __________________________ |
| --- | --- |

1. **How long have you been in this role?**

_______________________________

1. **What are your main responsibilities in this role?**

______________________________________________________________________________________________________________________________________________________________________________________________________________________________

__________________________________________________________________________

1. **Can you please decribe your level of involvement and responsibilities with regards to:**
   1. **The National Immunization Program**

______________________________________________________________________________________________________________________________________________________________________________________________________________________________

- 1. **Strategies for prevention, surveillance and treatment of childhood pneumonia**

______________________________________________________________________________________________________________________________________________________________________________________________________________________________

Perceptions of Pneumococcal disease burden

1. **Could you provide an overview of the burden of pneumococcal disease in your country?**

*[Probe: Perception of severity? Perception of susceptibility? Distinction from viral pneumonia?]*

______________________________________________________________________________________________________________________________________________________________________________________________________________________________

1. **What types of surveillance systems and data sources are generally used to track pneumococcal disease incidence and prevalence?**

______________________________________________________________________________________________________________________________________________________________________________________________________________________________

Perceptions of Pneumococcal disease susceptibility

1. **Are there specific pockets or populations that are more at-risk for pneumococcal disease? What are some contributors to their higher risk?**

______________________________________________________________________________________________________________________________________________________________________________________________________________________________

1. **What social, environmental or other factors do you believe contribute to pneumococcal disease occurance?**

______________________________________________________________________________________________________________________________________________________________________________________________________________________________

Perceptions of Health Systems Priorities

1. **What are some of the government/ MoH’s top national level priorities or targets to combat repisratory diseases in the upcoming years?**

____________________________________________________________________________________________________________________________________________________

1. **How would you describe the impact of pneumonia disease management on health systems in your country?**

______________________________________________________________________________________________________________________________________________________________________________________________________________________________

Strategies to Promote Vaccination

1. **How do you think PCV introduction may impact childhood health in your country?**

[*Probe*: Impact on mortality? Impact on morbidity? Impact on economy?]

______________________________________________________________________________________________________________________________________________________________________________________________________________________________

1. **What do you think about PCV introduction the national immunization program?**

______________________________________________________________________________________________________________________________________________________________________________________________________________________________

1. **What specific regions or populations do you believe should be prioritized during PCV introduction?**

______________________________________________________________________________________________________________________________________________________________________________________________________________________________

1. **I will now request you to reflect on potential challenges that may arise during a national level PCV roll out and suggest some strategies from your experience that may help to overcome them:**
   1. **What support or resources would be required to overcome challenges with supply chains or other logistics?**

______________________________________________________________________________________________________________________________________________________________________________________________________________________________

- 1. **What are some of the legal or regulatory challenges to PCV introduction in your country? What support would be required to address these?**

______________________________________________________________________________________________________________________________________________________________________________________________________________________________

1. **Please describe your experiences or takeways from other vaccine introductions in your country. How would you suggest applying these lessons to the context of PCV introduction?**

______________________________________________________________________________________________________________________________________________________________________________________________________________________________

Vaccine uptake and acceptance

1. **Which strategies would you recommend for PCV rollout in children?**

[*Probe*: texting campaign; social media; health communication efforts]

______________________________________________________________________________________________________________________________________________________________________________________________________________________________

1. **What factors do you think may lead to hesitancy or limited uptake for PCV for children among caregivers?**

[*Probe*: Lack of information; side effects; distrust; rumors; finances]

______________________________________________________________________________________________________________________________________________________________________________________________________________________________

1. **Where do people in your country get their information on vaccinations?**

[*Probe*: radio; televesion; print media; social media; health workers; community; family; friends]

______________________________________________________________________________________________________________________________________________________________________________________________________________________________

1. **What source of information do you think people find most reliable for vaccinations? Why?**

[*Probe*: radio; televesion; print media; social media; health workers; community; family; friends]

______________________________________________________________________________________________________________________________________________________________________________________________________________________________

1. **Are there any final thoughts or comments you would like to share?**

______________________________________________________________________________________________________________________________________________________________________________________________________________________________

**End of Interview. Thank participant for their time**
