# Supplementary material for: Bridging the gap in pneumonia prevention: Qualitative insights on vaccine implementation from health leaders in middle-income countries
Source: PLOS Glob Public Health. 2025 Apr 23;5(4):e0004473. doi: 10.1371/journal.pgph.0004473 (PMC12017571; doi:10.1371/journal.pgph.0004473)
Supplement: S1 Table — (DOCX) [file pgph.0004473.s002.docx]

**Interviewee Code**: _________

**Summary *(In this section, please include a summary statement, as well as any quotes, that summarize the largest takeaway messages from the interview)***

- Takeaway Point(s):
- Summary Quote(s):

| **Code / Key Theme** | **Subcode** | **Definition** | **Quote** | **Summary** |
| --- | --- | --- | --- | --- |
| Middle-Income Pricing / Costing | Unique Barriers | In this section, please document any barriers that individuals shared which are unique to MICs. |  |  |
|  | Price Negotiations | In this section, please document any discussions of how the country has pursued negotiating the cost of PCV, or how they intend to. |  |  |
|  | External Support | In this section, please document which internal or external agencies are assisting or hindering price discussions. |  |  |
| Alternative Priorities | Non-Communicable Diseases | In this section, please document if the country is prioritizing NCDs such as hypertension. |  |  |
|  | Vaccine-Preventable Diseases | In this section, please document if the country is prioritizing vaccines, or treatments for, other VPDs prior to PCV |  |  |
|  | Road Accidents | In this section, please document if the country is prioritizing road accidents. |  |  |
|  | Other Priorities | In this section, please document if the country is prioritizing anything else prior to PCV. |  |  |
| Communication  Strategies | Hesitancy | In this section, please document if the country faces hesitancy which they believe will need specific communication strategies. |  |  |
|  | Hard-to-reach populations | In this section, please document if the country has hard-to-reach populations which they believe will need specific communication strategies. |  |  |
|  | Zero Dose | In this section, please document if the country faces hesitancy which they believe will need specific communication strategies. |  |  |
| Introduction Preparation | Processes | In this section, please document the processes that need to be taken prior to PCV introduction. |  |  |
|  | Outstanding Needs | In this section, please document any outstanding needs to be addressed before introduction (i.e. training). |  |  |
| Surveillance | Formal Tracking | In this section, please document if the country has a formal surveillance method for pneumonia / pneumococcal pneumonia. |  |  |
|  | Serotyping | In this section, please document if the country has a formal serotyping approach for all (or a subset of) pneumonia cases. |  |  |
|  | Burden of Pneumonia | In this section, please document if the country has a formal surveillance method that allows them to capture the burden of pneumonia across the country. |  |  |
| Equity | Equity Research | In this section, please document if the country has led research about equity in vaccine access. |  |  |
|  | Equity in Communication | In this section, please document if equity angles will be used in communication with decision makers |  |  |
| NEW THEMES  Please add new themes as appropriate | | | | |
| New Theme #1 |  |  |  |  |
| New Theme #2 |  |  |  |  |
| New Theme #3 |  |  |  |  |
| New Theme #4 |  |  |  |  |
